# Aspects of Benthic Decapod Diversity and Distribution from Rocky Nearshore Habitat at Geographically Widely Dispersed Sites

**DOI:** 10.1371/journal.pone.0018606

**Published:** 2011-04-14

**Authors:** Gerhard Pohle, Katrin Iken, K. Robert Clarke, Thomas Trott, Brenda Konar, Juan José Cruz-Motta, Melisa Wong, Lisandro Benedetti-Cecchi, Angela Mead, Patricia Miloslavich, Nova Mieszkowska, Rebecca Milne, Laura Tamburello, Ann Knowlton, Edward Kimani, Yoshihisa Shirayama

**Affiliations:** 1 Atlantic Reference Centre, Huntsman Marine Science Centre, St. Andrews, New Brunswick, Canada; 2 School of Fisheries and Ocean Sciences, University of Alaska Fairbanks, Fairbanks, Alaska, United States of America; 3 Plymouth Marine Laboratory, Prospect Place, West Hoe, Plymouth, United Kingdom; Department of Biology, Suffolk University, Boston, Massachusetts, United States of America; 5 Departamento de Estudios Ambientales, Centro de Biodiversidad Marina, Universidad Simón Bolívar, Caracas, Venezuela; 6 Bedford Institute of Oceanography, Dartmouth, Nova Scotia, Canada; 7 Department of Biology, University of Pisa, CoNISMa (National Interuniversity Consortium of Marine Sciences), Pisa, Italy; 8 Department of Zoology, University of Cape Town, Cape Town, South Africa; 9 Marine Biological Association of the United Kingdom, Plymouth, United Kingdom; 10 Kenya Marine and Fisheries Research Institute, Mombasa, Kenya; 11 Seto Marine Biological Laboratory, Kyoto University, Wakayama, Japan; National Institute of Water & Atmospheric Research, New Zealand

## Abstract

Relationships of diversity, distribution and abundance of benthic decapods in intertidal and shallow subtidal waters to 10 m depth are explored based on data obtained using a standardized protocol of globally-distributed samples. Results indicate that decapod species richness overall is low within the nearshore, typically ranging from one to six taxa per site (mean = 4.5). Regionally the Gulf of Alaska decapod crustacean community structure was distinguishable by depth, multivariate analysis indicating increasing change with depth, where assemblages of the high and mid tide, low tide and 1 m, and 5 and 10 m strata formed three distinct groups. Univariate analysis showed species richness increasing from the high intertidal zone to 1 m subtidally, with distinct depth preferences among the 23 species. A similar depth trend but with peak richness at 5 m was observed when all global data were combined. Analysis of latitudinal trends, confined by data limitations, was equivocal on a global scale. While significant latitudinal differences existed in community structure among ecoregions, a semi-linear trend in changing community structure from the Arctic to lower latitudes did not hold when including tropical results. Among boreal regions the Canadian Atlantic was relatively species poor compared to the Gulf of Alaska, whereas the Caribbean and Sea of Japan appeared to be species hot spots. While species poor, samples from the Canadian Atlantic were the most diverse at the higher infraordinal level. Linking 11 environmental variables available for all sites to the best fit family-based biotic pattern showed a significant relationship, with the single best explanatory variable being the level of organic pollution and the best combination overall being organic pollution and primary productivity. While data limitations restrict conclusions in a global context, results are seen as a first-cut contribution useful in generating discussion and more in-depth work in the still poorly understood field of biodiversity distribution.

## Introduction

With biological diversity generally recognized as providing the variability essential to cope with changes in nature [1]–[2] and loss of biodiversity impairing ecosystem goods and services [3]–[4], the severe depletion in populations or entire loss of species in recent decades has brought the importance of biodiversity to the forefront [5]–[6]. While there is a growing body of literature on the subject, estimates of the number of marine species still vary widely [7]–[8] and large gaps remain in the understanding of how species are distributed in space and time [9] or how they contribute to ecosystem processes [10]–[11]. Thus one of the most important objectives in ecology and biogeography remains the “development of a markedly improved understanding of the global distribution of biodiversity” [12].

Marine systems have for some time been regarded as less impacted than freshwater or terrestrial counterparts because of fewer documented losses of species and a perceived “extra measure of resilience” because of larger geographic ranges [5] and greater phyletic diversity [7], [13]. However, not only may marine extinctions be underestimated [14] but recent evidence also indicates widespread changes in biodiversity in terms of the composition and abundance of species. This includes the severe depletion of large fish populations [15]–[16] that amount to ecological extinction [17], the decline of corals [18], and the spread of invasive species [19] that are visible danger signs of substantial changes occurring within the marine realm on a global scale.

The understanding of biodiversity in coastal regions is of particular importance as it is where the majority of the human population now lives and where demographics project that 75% of humanity, or 6.4 billion people, will reside within three decades [20]. This represents more people than the current global population. Loss of marine diversity is highest in the coastal zone [21], where habitats have been and are being profoundly altered [4], [9], with more dramatic changes likely in the future. An evaluation of the scale and consequences of these changes is hampered by a lack of basic knowledge of the diversity, patterns and processes that control the distribution and abundance of organisms within coastal areas.

The intent of this paper is to compare and contrast diversity and abundance of benthic crustacean decapods from intertidal and shallow subtidal rocky shore sites at geographically widely dispersed areas to ascertain if there are trends with depth and latitude and what environmental drivers may explain patterns in decapod assemblages.

## Methods

Decapod diversity data were obtained based on specimens collected using a standardized protocol [22] developed for the Census of Marine Life NaGISA program (Natural Geography in Shore Areas, www.coml.nagisa.org) for coastal hard substrate sites with macroalgal cover, from here on referred to as rocky shores. The protocol is based on the use of 30 m transect lines set parallel to the shore, at three intertidal levels (high, mid and low) and three subtidal depths (1, 5 and 10 m), using 25×25 cm and 1×1 m quadrats at each of 5 randomly selected replicate stations per transect. The two quadrat sizes were employed to account for possible scaling effects and to use as a comparable tool for a like-with-like comparison, but not to estimate the maximum or actual total diversity.

Data on decapod taxa and abundance were obtained from 36 sites (Supplementary Table S1) sampled once between 2005 and 2009, with the exception of earlier sampling of Alaska sites (2003) and repeated sampling of two sites in the NW Atlantic Fundy region (2007–2008). Generally field work was undertaken during the season of highest diversity and abundance. Limitations within the data set include the restricted number of sites, the absence of data from certain critical regions, (e.g. Antarctic and Australia) that were not covered and the limited and/or uneven number of sites per ecoregion. These limitations prevented an in-depth global scale analysis as originally intended, instead resulting in a restricted comparison of point species richness of limited regions. With only three of 36 sites from the southern hemisphere (Argentina and Africa) that sector could not be analyzed separately from the northern hemisphere, leading to the restriction of the latitudinal analysis to the northern hemisphere even though there is some indication of symmetrical hemisphere distribution of decapods [23]. For a considerable number of sampled sites decapod data were not recorded, or only at a coarser than species taxonomic level. Other regions were unable to obtain data for some tidal levels or not at the full level of replication. Sampled decapods fell into large and small size groups. While these were represented in 1 m^2^ and 0.0625 m^2^ quadrats, respectively, certain regions were predominated by one (e.g. small hermit crabs in Gulf of Alaska) or the other (e.g. large brachyuran crabs in Canadian Atlantic). This, and not all regions having sampled with both quadrat sizes, precluded intersite comparisons for each quadrat size, requiring adjustment of abundance data from the smaller to larger quadrat for analyses that included an abundance component. Alternatively, data were analyzed based on absence/presence.

Statistical analyses were undertaken using PRIMER 6 v. 6.1.13 (PRIMER-E Ltd.) with PERMANOVA+ for PRIMER and Statgraphics Centurion XV (StatPoint Inc.) for multi- and univariate components, respectively. Both classic univariate techniques and non-metric multivariate methods, which better cope with our specific data short comings, were employed for analysis. In order to mitigate the various limitations for comparative use globally, replicates of samples were averaged for a particular site and depth. For multivariate analysis this was followed by standardizing the pooled samples by their totals, resulting in totals that add up to 100 across a set of species and therefore indicating how percent composition changes by locality or depth. To balance contributions of abundant and rarer taxa, data were moderately (square root) transformed given the modest presence of rare species, followed by calculation of the Bray-Curtis coefficient to summarize the overall similarity between any samples (by taking all taxa and their relative abundance into consideration) for use in multivariate non-metric MDS ordination analysis.

From the composition of taxa it was apparent that, other than for adjacent sites, few taxa are shared between geographically widely separated sites, resulting in degenerate artefactual solutions [24] of the MDS analysis, as evidenced by collapsed Shepard plots. This was handled in two ways, one being the analysis of subsets of species data from restricted geographic areas, but thereby limiting analysis to few ecoregions, such as the Gulf of Alaska.

Alternatively, to enable a wider scale comparison, data were brought onto a more common footing. This was undertaken in two ways, firstly by aggregating data to the higher taxonomic family level where, in contrast to species and genera, families are shared in common amongst more widely separated regions. Recognizing that use of the three depths groups, as per Alaska depth results, generated degenerate plots, site data were also averaged for depth, allowing the use of sites as the replication level. While this resulted in non-degenerate plots the results may be compromised by the fact that depth ranges are not equally represented at each site. However, depth effects were deemed small in comparison to region effects (see results).

Secondly, a taxonomic dissimilarity measure based on the taxonomic distinctness concept, that incorporates relative relatedness of species and avoids aggregating to higher taxonomic levels, was employed. The dissimilarity coefficient Gamma^+^ [25] was produced for ordinating samples based instead on presence/absence data that mitigate a possible abundance scaling issue due to two different quadrat sizes used. This procedure is deemed of particular use where data are from widely separated regions that have few species in common. By way of including the different depth strata, this approach also permitted two-way ANOSIM testing in a crossed layout with six depths and eleven regions as factors to ascertain relative contributions of these two factors and to discern possible depth groupings.

Latitudinal trends analysis of species richness and interaction with other factors involved the use of PERMANOVA analysis of variance permutation testing using Euclidean distance, i.e. a standard ANOVA table but testing carried out by permutation rather than normality assumptions [26]. This is based on (univariate) species richness per individual quadrat, averaged over replicates for each site by depth combination and then log transformed to avoid the standard problem that pooling unequal numbers of replicates will strongly bias the resulting richness values. A second analysis used average taxonomic distance (Δ+) from species lists in pooled replicates since, unlike S, values of Δ+ are independent of sample size, so pooling unequal numbers of samples before its calculation will not bias the outcome [27].

Linking of community assemblage to environmental measures was undertaken to find the best match between the multivariate among-sample patterns and environmental variables, where the extent of pattern matching reflects the degree to which environmental data ‘explain’ the biotic pattern using both BEST and RELATE analyses [28]. Among a data set of 15 environmental variables [29], 11 with complete information were used in the global BEST analysis (Table 1). These 11 variables included a set of three natural environmental drivers, consisting of sea surface temperature (SST [30]), chlorophyll-*a* density (CHA [31]), and primary productivity (PP [32]), derived as per Benedetti-Cecchi et al. [29]. The remaining eight indices of anthropogenic variables included inorganic pollution (INP), organic pollution (ORP), nutrient contamination (NUTC), marine-derived pollution (MARP), acidification (AC), invasive species incidence (INV), human coastal population density (HUM), and shipping activity (SH), taken from 1 km resolution global models of human impacts on marine ecosystems [33]. Gulf of Alaska samples were taken at all depth strata so these depths (scaled as 1–6 for high intertidal to the 10 m stratum) were utilized separately in the RELATE regional analysis for those samples.

**Table 1:** List of natural and anthropogenic environmental variables used in global (*) and Gulf of Alaska (^+^) regional analyses.

Variable	Short	Description	Reference
**Natural**			
Sea-surface temperature*	SST	Climatological summer mean value, averaged between 1985 and 2001, derived from the 4 km resolution AVHRR Pathfinder Project version 5.0 by the NOAA NODC	[30]
Chlorophyll-a*	CHA	SeaWiFS reprocessing 5.2 by the NASA GSFC Ocean Color Group, averaged 1997–2009, 9 km resolution	[31]
Primary productivity*	PP	mg carbon m^−2^ d^−1^, Vertically Generalized Production Model (VGPM) for SeaWiFS, averaged 1997–2007, 18 km resolution	[32]
Depth^+^	D	High intertidal to 10 m	NaGISA data
**Anthropogenic**			
Inorganic pollution*	INP	Urban runoff estimated from land-use categories, US Geologic Survey (http://edcsns17.cr.usgs.gov/glcc/)	[33]
Organic pollution*	ORP	FAO national pesticides statistics (1992–2002), (http://faostat.fao.org)	[33]
Nutrient contamination*	NUTC	FAO national fertilizers statistics (1992–2002), (http://faostat.fao.org)	[33]
Marine-derived pollution*	MARP	Port data 1999–2005, proportional to commercial shipping traffic	[31]
Acidification*	AC	Aragonite saturation state 1870–2000/2009	[33]
Invasive species incidence*	INV	Cargo traffic 1999–2003	[31]
human coastal population density*	HUM	LandScan 30 arc-second population data of 2005	[33]
shipping activity*	SH	Commercial ship traffic 2004–2005	[33]

Based on an assessment of pair-wise scatter plots of all variables for global analysis, eight of 11 variables (AC, CHA, INP, INV, NUTC, ORP, PP, SH) were selectively log transformed (log (0.1 + y)) to approximate a symmetric and linear distribution of data points, with all variables then normalized (value less mean, divided by standard deviation for each variable) to bring values of the various variables onto a common and comparable dimensionless measurement scale. Examination for correlation prior to analysis using Spearman Rank correlations revealed that none of the variables were correlated at rho ≥0.95 and thus all variables were maintained in the analysis.

Community structure data, used for comparison with environmental results, were based on family-level aggregation of original species data that were standardized, square root transformed and depth averaged for all sites. The environmental and biotic matrices were analyzed using the BEST routine, based on Euclidian distance and Bray-Curtis dissimilarity measures, respectively, to find the best match environmental variables using the Spearman rank correlation method that measures agreement between the two matrices. The results are then listed in terms of the best combination for a fixed number of explanatory variables, up to the point at which the values of the Spearman correlation start to fall below that for the optimum combination. This gives an indication of which are the most important environmental variables implicated in the (correlative) ‘explanation’ of the biotic structure. A major concern of this biological-environmental comparison was that both data sets were collected at different spatial resolution, especially with satellite-derived data that were obtained on a much coarser scale. Other environmental data were collected at a 1 km resolution but from global models that do not capture the particularly high variability of the nearshore system from where our biological data originated. Hence, for global comparison, biological and environmental data were averaged by ecoregions for the analysis to minimize small-scale biological variability and to scale up to the coarser resolution of environmental data (see [34] for more details). This procedure could potentially introduce some aggregation error, and that possibility is addressed in the interpretation of results.

## Results

**Figure 1:**
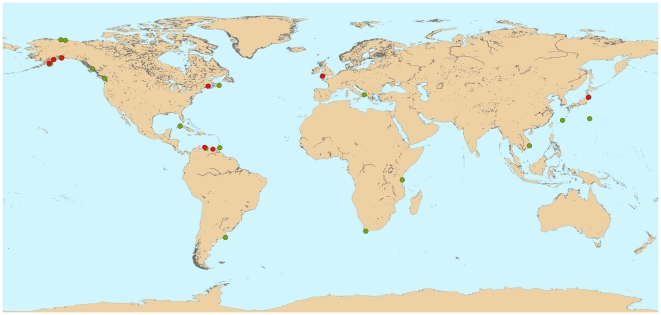
Global distribution of sampling sites with decapod crustacean assemblages within the NaGISA Census of Marine Life program. Sites that overlap at this scale are shown in red over green; see supplementary Table S1 for more details.

A total of 82 decapod taxa were identified from 293 sample quadrats analyzed for 36 sites from approximately 70°N to 38°S latitude (Fig. 1.), broadly representing eleven zoogeographic provinces (Supplementary Table S1), that, with few exceptions, are similar to the ecoregions of Iken et al. [32], or some of the 64 large marine ecosystems (LME) of Sherman et al. [35]. In general decapod diversity was quite low, ranging from one to 13 species per site (mean = 4.5), with a typical range of one to six species for two thirds of all sites.

### Depth trends

**Figure 2:**
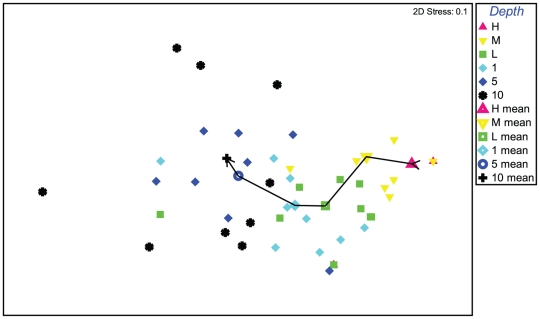
Multivariate analysis nMDS plot of depth-related decapod crustacean assemblage structure from the nearshore Alaskan Pacific. Based on species abundance data and Bray-Curtis similarity measure; individual sites (solid symbols, each representing five replicates) and mean of all sites (open symbols) are displayed by six depth intervals; line indicates progression from high intertidal to 10 m subtidal stratum.

**Figure 3:**
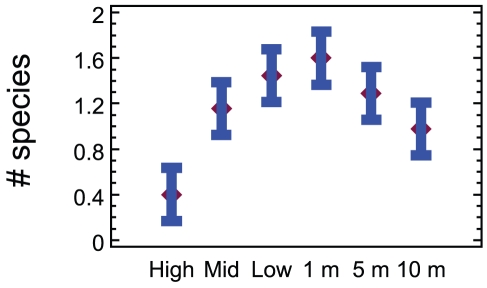
Decapod species richness on rocky shore in the Gulf of Alaska. Mean and 95% confidence intervals per 0.0625 m^2^ at high intertidal to 10 m subtidal strata; diversity at the high intertidal stratum is significantly lower than all other strata, as is the 10 m stratum compared to low tide and 1 m strata (P<5%); n = 45 quadrat records per depth stratum.

Multivariate analysis of regional data subset samples from the Gulf of Alaska, all based on 25×25 cm quadrats, showed a distinct and quasi-linear depth trend with an increasingly changing assemblage structure from the high tide level to 10 m subtidally (Fig. 2). Matching ANOSIM testing confirms this with higher R statistics the further apart two depth groups are (Table 2), with high and mid tide, low tide and 1m, and 5 and 10m representing levels that are not significantly different among each pair (p > 10.4%) but the three pairs forming significantly different groups (p<2.8%). Univariate results indicate that diversity changed in terms of depth, with lowest species richness at the high intertidal substratum, increasing to a peak at 1 m, then decreasing to 10 m (Fig. 3). Depth preferences among the 23 species of anomuran, brachyuran and caridean decapods were also discernible (Table 3) as follows: (1) no single species occurred at all strata, (2) unlike brachyuran and anomuran taxa, all six shrimp species were restricted to the subtidal zone, with the exception of a single record of one species, (3), the hermit crab *Pagurus hirsutiusculus* showed a strong preference for the intertidal zone, with peak abundance at the high intertidal stratum, where no other decapods were found, (4) while four taxa, the anomurans *Pagurus beringanus*, *P. caurinus* and brachyurans *Cancer oregonensis* and *Pugettia gracilis*, occured in five of the six depth strata, depth preferences could be discerned in terms of abundance peaks at 1 and 5 m strata, and (5) all but one of the eight remaining anomuran pagurids were restricted to the subtidal with peak abundances at 5 m for all but two species.

**Table 2:** Analysis of similarity for Gulf of Alaska decapod assemblage structure depth pattern based on site data and depth groupings as per Figure 2.

Global test					
Sample statistic (Global R): 0.388					
Significance level of sample statistic: 0.01%					
Number of permutations: 9999 (Random sample from a large number)					
Number of permuted statistics greater than or equal to Global R: 0					

Pairwise Tests					
	R	Significance	Possible	Actual	Number > =
Groups	Statistic	Level %	Permutations	Permutations	Observed
H, M	0.055	10.4	24310	9999	1043
H,L	0.474	0.1	24310	9999	9
H, 1	0.680	0.04	24310	9999	3
H, 5	0.752	0.03	6435	6435	2
H, 10	0.604	0.05	24310	9999	4
M, L	0.340	0.1	24310	9999	13
M, 1	0.586	0.03	24310	9999	2
M, 5	0.703	0.02	24310	9999	1
M, 10	0.567	0.01	24310	9999	0
L, 1	0.045	20.9	24310	9999	2086
L, 5	0.366	0.6	24310	9999	63
L, 10	0.341	0.1	24310	9999	11
1, 5	0.228	2.8	24310	9999	279
1, 10	0.271	0.3	24310	9999	30
5, 10	−0.052	72.7	24310	9999	7270

**Table 3:** Relative abundance of 23 decapod species among six depth strata recorded from nine Gulf of Alaska ecoregion sites in 2003.

Species	High tide	Mid tide	Low tide	1 m	5 m	10 m
Anomura, Paguridae:						
*Pagurus hirsutiusculus*	109	91	64	12	0	0
*Pagurus beringanus*	0	35	31	39	20	6
*Pagurus caurinus*	0	1	6	17	32	20
*Pagurus granosimanus*	0	2	0	0	0	0
*Elassochirus gilli*	0	2	0	3	5	2
*Elassochirus cavimanus*	0	0	0	1	2	0
*Elassochirus tenuimanus*	0	0	0	0	7	2
*Pagurus dalli*	0	0	0	0	1	3
*Pagurus kennerlyi*	0	0	0	0	6	8
*Pagurus* sp. A	0	0	0	0	1	0
*Discopagurus schmitti*	0	0	0	0	2	0
Anomura, Lithodidae:						
*Cryptolithodes* sp.	0	1	2	4	1	0
Brachyura, Atelecyclidae:						
*Telmessus cheiragonus*	0	0	3	1	0	1
Brachyura, Majidea:						
*Pugettia gracilis*	0	27	113	199	14	14
*Oregonia gracilis*	0	0	7	1	0	0
Brachyura, Cancridae:						0
*Cancer oregonensis*	0	12	8	24	3	2
Caridea, Hippolytidae:						
*Eualus* sp.	0	0	0	0	0	2
*Heptacarpus herdmani*	0	0	0	0	1	0
*Heptacarpus puggettensis*	0	0	0	3	0	0
*Heptacarpus sitchensis*	0	0	0	0	0	1
*Hippolyte clarki*	0	1	0	0	0	1
*Lebbeus grandimana*	0	0	0	0	0	1
*Spirontocaris snyderi*	0	0	0	0	3	5

A similarly clear depth trend was not detected for other ecoregions but when samples from all regions were combined diversity peaked at 5 rather than 1 m (data not shown), otherwise agreeing with the depth pattern of the Gulf of Alaska. Lack of shared taxa did not permit a global taxonomic depth characterization.

### Latitudinal trends

Multivariate analysis, based on the Gamma+ resemblance measure, showed no distinct latitudinal pattern when separated into three depth groups as per depth analysis (Supplementary Fig. S1). Two-way ANISOM testing of all data in a crossed layout, with all depths and regions as factors, yielded significant global R statistics (p<0.1%) that indicate differences among regions and depth strata. However, the global R value of 0.645 for regions was substantially higher than 0.213 for depths, indicating that assemblages by regions are much more separated, and therefore different from each other, than are depth groups. This is supported by Gamma+ plots, showing that while assemblages tended to cluster by region (Supplementary Fig. S2A), they did not form groups by depths strata (Supplementary Fig. S2B).

**Figure 4:**
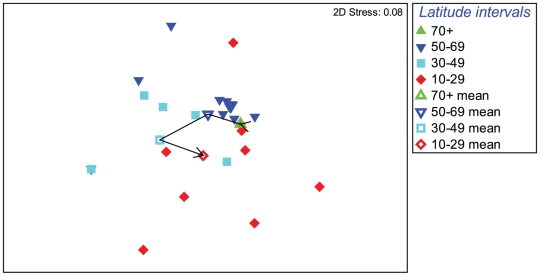
Non-parametric multivariate analysis MDS plot of global-scale decapod assemblage structure. Based on family-level aggregation of depth-averaged species data, in terms of latitudinal distribution for individual sites (closed symbols) and mean of all sites (open symbols) per latitudinal interval.

With depth differences in assemblage structure deemed smaller than those between regions, averaging depths was deemed justified to allow use of sites as the replication level, thus maximizing available site data. Consequential analysis with depth averaged data aggregated to family level, based on the Bray-Curtis similarity measure, showed that significant latitudinal differences existed in community structure among geographic regions (global test R = 0.16, p<0.8%). As indicated by mean values of latitude intervals (Fig. 4), a semi-linear trend of increasingly changing assemblages was evident from polar latitudes to the 30-49° temperate interval, with significant differences between assemblages of subpolar and temperate regions (p<0.7%). However, this trend did not apply to tropical results, shown to be significantly different from subpolar (p<0.4%) but not from temperate regions (p = 18.8%). R statistics of pairwise tests indicated the strongest separation between the 30–49° and 50–69° latitude groups.

**Figure 5:**
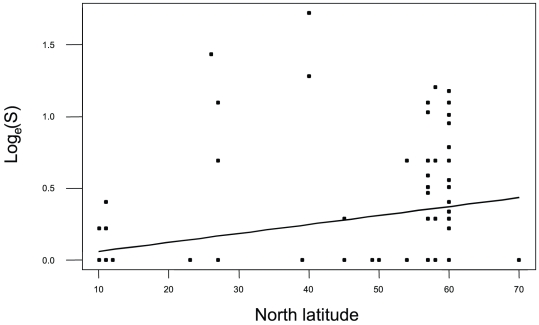
Regression plot of decapod species richness against latitude for data from all sites and depths. Log_e_(S)  =  −0.003+0.0063 x latitude (F = 8.6, p = 0.4%).

While skewed and high in variance, a plot of species richness against latitude for all data combined suggests a positive relationship of richness to latitude (Fig. 5). Analysis of covariance factor permutation testing to ascertain whether richness was a function of depth and/or latitude, indicated a significant relationship between richness and latitude, and an intertidal versus subtidal effect (Table 4). The order of inclusion of terms did not materially change these conclusions since there was little relation between depth and latitude. Resulting separate intertidal and subtidal plots (Supplementary Fig. S3) indicate this by a difference in intercept and the same slope, respectively. An analogous covariance analysis using average taxonomic distance (Δ+) showed only a marginal relationship of Δ+ to latitude (p = 6.7%) but an interaction between latitude and differences between intertidal and subtidal divisions (F =  15.2, p = 0.2%, Table 5). When intertidal and subtidal zones were analyzed separately (Table 6) this was manifested by a significant relationship between Δ+ diversity and the intertidal (F = 16.6, p = 0.2%) but not with the subtidal (p = 31%).

**Table 4:** PERMANOVA analysis of covariance permutation testing using Euclidean distance, showing results of log(S) correlation with latitude, intertidal versus subtidal difference (IT vs ST), depth differences within IT or ST (De (IT vs ST)), latitude interaction within intertidal or subtidal (La x IT vs ST)), and latitude interaction within the six depth strata (La x De (IT vs ST)).

Source	df	SS	MS	F	P value	Unique per- mutations
**Latitude**	**1**	**1.4975**	**1.4975**	**10.078**	**0.006**	996
**IT vs ST**	**1**	**1.1358**	**1.1358**	**7.644**	**0.009**	998
De (IT vs ST)	6	0.96431	0.16072	1.0816	0.365	998
La x IT vs ST	1	1.42E-02	1.42E-02	9.59E-02	0.788	998
La x De (IT vs ST)	5	0.7993	0.15986	1.0758	0.338	999
Residual	104	15.453	0.14859			
Total	118	19.865				

Significant differences (p<0.05) are in bold.

**Table 5:** Results of PERMANOVA to test for taxonomic distinctness (Δ+) relation with latitude, intertidal versus subtidal difference (IT vs ST), depth differences within IT or ST (De (IT vs ST)), latitude interaction within intertidal or subtidal (La x IT vs ST)), and latitude interaction within the six depth strata (La x De (IT vs ST)).

Source	df	SS	MS	F	P value	Unique perms
Latitude	1	988.5	988.5	3.2481	0.067	997
IT vs ST	1	56.694	56.694	0.1863	0.673	997
De (IT vs IT)	5	2148.2	429.63	1.4117	0.238	998
**La x IT vs ST**	**1**	**4637.5**	**4637.5**	**15.238**	**0.002**	997
La x De (IT vs ST)	5	1985.6	397.13	1.3049	0.274	999
Residual	47	14304	304.33			
Total	60	24120				

Significant differences (p<5%) are in bold.

**Table 6:** Results of PERMANOVA to test for taxonomic distinctness (Δ+) relation with latitude and depth, and their interaction, carried out separately for intertidal and subtidal samples.

IT only						
Source	df	SS	MS	F	P(perm)	Unique perms
**La**	**1**	**3958.6**	**3958.6**	**16.652**	**0.002**	996
De	3	1639.8	546.58	2.2992	0.139	999
La x De	3	1459.4	486.48	2.0464	0.215	998
Residual	16	3803.6	237.72			
Total	23	10861				

ST only						
Source	df	SS	MS	F	P(perm)	Unique perms
La	1	368.14	368.14	1.0869	0.314	996
De	2	1850.6	925.3	2.7318	0.089	999
La x De	2	526.2	263.1	0.77677	0.478	999
Residual	31	10500	338.71			
Total	36	13245				

Significant differences (p<5%) are in bold. Abbreviations as per table 4.

Four major groups of decapods were represented among the entire data set (Table 7), with anomuran and brachyuran crabs being represented in most, and caridean shrimps at about half of the regions, while lobsters were recorded only from the Canadian Atlantic. The latter ecoregion was the only one with representation from all four groups. However, in terms of lower taxa this region is relatively depauperate, despite the largest number of samples taken amongst all regions (incl. consecutive years). In comparison, the even more northerly American west coast Gulf of Alaska ecoregion is represented by three times as many decapods than the Canadian Atlantic coast, with lithodid and pagurid anomurans comprising the majority of decapods. Other decapod hot spots appear to be the Caribbean Sea and waters surrounding Japan, the latter despite a relatively low number of samples. Results from other ecoregions should be considered as preliminary as they may not be indicative due to a relatively low number of samples or sites.

**Table 7:** Number of species^1^ recorded among four major decapod groups for 11 sampled eco-regions.

	Arctic	Alaska	BC*	UK	Can. Atl.	Med.	Argen-tina	Africa†	Japan	Vietnam	Carib-bean
Latitude (approx.)	70	60	50	50	45	40	−40	−35, −6	30	15	15
N samples	25	270	50	60	360	40	50	30	25	6	200
**Anomura** (false crabs)	1	12	1	0	1	3	1	0	2	0	13
**Brachyura** (true crabs)	0	4	2	3	3	3	2	3	7	2	5
**Caridea** (shrimps)	0	7	0	0	2	3	0	0	4	0	1
**Astacidea** (lobsters)	0	0	0	0	1	0	0	0	0	0	0
Total species^1^	1	23	3	3	7	9	3	3	13	2	19

BC  =  British Columbia; Can. Atl.  =  Canadian Atlantic; UK, United Kingdom; Med. =  Mediterranean.

*, often considered the southern limit of “Alaska” ecoregion;

† , data from two sites at zoogeographically disjunct regions combined for inclusion purposes only;

1 , for some ecoregions may include specimens originally identified to a shared higher taxon, but here assumed to represent distinct species among ecoregions.

### Correlation with environmental drivers

The BEST procedure, used to link 11 environmental variables available for all sites (Table 8) to the family-based biotic pattern, showed a significant relationship between the community structure and environmental variables (Rho = 0.70; p<3%) on the global scale. The best correlation match (Rho = 0.70) consists of just two variables, organic pollution and primary productivity (Table 8), with the highest individual contributor being the former (Rho = 0.45). Three other combinations involving up to three additional variables result in lower than optimal (Rho = 0.65–0.68) but similar correlation values (Table 8). Regionally, within the Gulf of Alaska, the RELATE test showed depth to be significantly related to the biotic pattern (Rho = 0.53; p<0.01%).

**Table 8:** Results of BEST Bio-Env global analysis indicating which environmental variables amongst a set of 11 best-match decapod assemblage structure similarity matrices.

Number of variables	Rho correlation	Best variable combination
12345	0.4520.7000 .6800.6630.646	Organic pollutionOrganic pollution, primary productivityOrganic pollution, primary productivity, shippingOrganic pollution, primary productivity,inorganic pollution,invasivesprimary productivity, inorganic pollution, invasives, shipping

## Discussion

In a global context this study encountered data limitations primarily in terms of geographical coverage, small sample sizes and uneven sampling effort in general. For example, four similar studies based on NaGISA samples [29], [34], [36]–[37], including different faunal and macroalgal components, were based on about twice the number of sampling sites as in the present study. Thus, while this restricted analysis and results should be considered preliminary on a global scale, they lend themselves to discussion and comparisons with other work in this field and as a stimulus to generate more in-depth work anticipated as more sampling occurs at existing and additional NaGISA sites.

### Depth stratification

A comparable global analysis of macroalgae [36] with overlap for sites of the present study shows mean taxonomic richness similarly increasing, progressing from the high intertidal to the sea and peaking at the 1 m stratum, but at a higher diversity level. Biomass also peaked at the 1 m stratum for macroalgae. A different rocky shore study that was restricted to the Gulf of Alaska [38] and included macroalgae, polychaetes, echinoderms and molluscs, concluded that, while these groups as a whole showed greatest richness at the low intertidal and 1 m depth strata, regional variation within and among different taxa prevented a generalization of trends for each of the four taxonomic groups across the region.

### Latitudinal trends (global)

While the dearth of comparable data in this study is a likely factor in not uncovering clear trends, standard multivariate ordination analysis was also complicated by the fact that few species were shared among widely separated regions on a global scale, creating a special case scenario requiring further manipulations, each with certain compromises (see methods). Most revealing was multivariate analysis using family level aggregation that indicated a progressive change in community structure from the Arctic to temperate latitudes, with significant differences between populations of subpolar and temperate regions. However, this trend did not continue for the tropical latitude interval. Univariate analysis showed no clear correlative latitudinal trend in species richness, with an indication of a possible increase in diversity with higher latitudes that is contrary to expectations ([23], see below). Small sample sizes and limited coverage precludes our results from being sufficient evidence one way or another for a latitudinal gradient. Clearly additional data points are needed to investigate this aspect further that, as pointed out by Gray [39], is a weakness inherent in a number of similar studies.

Surprisingly, while a gradient of decreasing species diversity with increasing latitude is well-established in terrestrial plant and animal communities, evidence in the sea is equivocal [39]–[42]. For example, no clear trend is evident among encrusting Bryozoa [43]. While an Arctic to tropics cline of decreasing species richness is evident for isopods, bivalves and gastropods from the deep-sea [44]–[45], there is evidence for at least two different trends among some shallow-water taxa: seaweeds indicate higher algal diversity in temperate areas, both from the NaGISA study [36] and other research [46]–[47], as do intertidal echinoderms collected in 25×25 cm quadrats [34]. In contrast, gastropod and bivalve molluscs, foraminiferans and reef-building corals exhibit greater species richness in the tropics [42], as is the case for most terrestrial communities. Interestingly, when NaGISA data on entire rocky shore communities were analyzed, no latitudinal trends were found [37], indicating that different taxa likely have different latitudinal responses that may be obscured in whole community analyses. This illustrates the importance of analyzing both individual taxonomic groups, as done for macroalgae, polychaetes, echinoderms and now decapods in the NaGISA program, as well as whole macroinvertebrate communities in specific habitats to gain a better understanding of latitudinal diversity patterns and drivers.

It has been postulated that marine animal groups with calcareous skeletons exhibit higher diversity at low latitudes, leading to the speculation that this latitudinal trend may be the result of an increasing thermodynamic cost in producing a calcium carbonate skeleton at decreasing temperatures of higher latitudes [48], and that this latitudinal cline of increasing diversity towards lower latitudes may be limited to taxa with calcareous skeletons. However, NaGISA results on echinoderms [34], that heavily depend on calcareous skeletal structures, appear to contravene this supposition.

Decapods, like all crustaceans, have an exoskeleton of chitin that also requires calcium for hardening, achieved by deposition of calcium salts in the organic matrix of the cuticle [49]. Evidence for latitudinal trends within that group indicates that within Pacific and Atlantic coastal waters (0–200 m) of the Americas [23] the distribution of decapods shows a larger diversity of species in tropical regions, decreasing gradually toward higher latitudes, a trend observed both among brachyurans and the rest of the decapods. However, a similar analysis of coastal (0–100 m) decapods in the western and eastern Atlantic [50] shows that gradients of benthic decapods are not symmetric for both coastlines, instead showing a single western diversity peak in the Caribbean at about 25°N, compared to two eastern peaks near the equator and at 35°N, the latter encompassing the Mediterranean. These differences are primarily attributed to coastal hydrographic processes.

A difficulty in understanding latitudinal trends is evidence that in general the Southern Ocean is strikingly more diverse in species than Arctic waters [51], differences in evolutionary history, climate and oceanography indicating that latitudinal trends differ in the northern and southern hemisphere at least in terms of polar diversity. This is contrary to evidence for a symmetrical trend of decapods in the two hemispheres for the Americas [23], which included an Arctic but no Antarctic component. However, Antarctic decapod species diversity is strikingly low compared to other groups [52], with brachyuran crabs and lobsters completely absent. Thus the trend of decreasing diversity with higher latitudes in the southern hemisphere may hold for decapods in particular.

Even though substantially different depth ranges are involved, results on relative taxonomic richness of the present study (Table 7) are consistent with those of Boschi [23] for coastal waters to 200–300 m depth in the Americas. Both studies found the lowest decapod species diversity was in the Arctic, followed by the “boreal Atlantic province”, equivalent to the Canadian Atlantic herein, with the diversity of the equivalent NE Pacific Gulf of Alaska “Aleutian province” being markedly higher than the boreal Atlantic counterpart. Similarly, the Caribbean is a decapod hot spot in both studies, as it is for terrestrial plants and vertebrates [53]. This region apparently is also a hot spot for echinoderms [34]. Taken together, “the assumption of a simple and universal latitudinal diversity cline in the sea is, on present evidence, probably unwarranted” [42] but also in need of much more investigation.

### Environmental variables

Based on an analysis of fishes and invertebrates from benthic and pelagic habitats Macpherson [50] concluded that no single primary “cause” or factor explains the pattern of marine species on a large spatial scale. In general, more than a single mechanism is likely involved that may vary with spatial scale [12]. Nevertheless, on a regional scale, a substantial proportion of species richness can apparently be explained statistically in terms of a few environmental variables [54], but not in the form of a predictive theory [12].

Results in our study indicate that on a regional scale depth is a significant factor explaining the community assemblage of benthic decapods in the nearshore environment. The need for depth averaging of assemblage data did not allow for a similar evaluation on a global scale, where primary productivity was the most important natural variable but a more anthropogenic measure of organic loading was the best single explanatory variable (Table 8). While aggregation error could confound conclusions drawn from such comparisons, a related NaGISA study on polychaetes [29] similarly found evidence that nutrient contamination and pollution can have an effect on such an assemblage at continental and intercontinental scales. Nevertheless, our results should be viewed with care given the biological and environmental data constraints (see methods) but may serve as a first approximation of which variables may be particularly important in decapod diversity. A lack of major depth effect would not be in conflict for the wider global picture, where some of the environmental variables may take very different ranges of values, and the more subtle, local depth effects become minor on that scale, as other results indicate in this study. Also, unlike the other environmental variables used in the global analysis, depth differs in representing more of an integrative measure that includes and reflects effects of several variables such as temperature, light intensity, salinity etc., thus preventing a straight comparison with other factors.

Presently the use of biodiversity in investigating ecosystem functional diversity is primarily in terms of relative taxon composition and trophic group analysis [55]. However, biological traits analysis [56] is a relatively new tool, where species diversity and biomass data are used in combination with information on key biological traits in ordination analysis to describe ecological functioning that should lead to new perspectives and would be a natural extension of studies such as the present one.

### Legacy and lessons learned

The use of a standardized protocol simple enough to be employed by diverse participant groups with varying expertise in different parts of the world has been the principle driving force in the realization of the NaGISA program. This is clear from continuing activities and the emergence of new sampling sites beyond the present study and by the fact that regional programs will continue beyond the present Census of Marine Life program. In the long term this may address the need for representation of unsampled regions and to obtain sample sizes appropriate for global comparison. Two primary procedural challenges emerged during the present study that should be better addressed in the future: one is the need for all of the components of the protocol to be completed at all sites, particularly sampling of all depth strata, replicates and target organisms. The other challenge is the need for taxonomic expertise that was not available in several regions. The former may be improved with better logistical support, the latter by having a central authority undertaking and/or coordinating identification-related activities rather than making this a regional responsibility. Without a doubt the primary lesson learned in terms of results is that much more comparable biological and environmental data are needed to establish clear patterns of distribution and environmental effects over large geographic scales and that one must be careful in generalizing trends as such simplifications invariably do not apply in the marine realm when considering different organismal groups in their specific habitats.

## Supporting Information

Figure S1 nMDS plots of global-scale decapod community structure. Based on presence/absence data and Gamma+ similarity measure, displayed for (A) high and mid intertidal, (B) low intertidal and 1 m, and (C) 5 and 10 m depth intervals at individual sites.
(EPS)

Figure S2 nMDS plots of global-scale decapod community structure. Based on presence/absence data and Gamma+ similarity measure, displayed by (A) region, and (B) six depth strata at individual sites.
(EPS)

Figure S3 Plots of log transformed decapod species richness against absolute latitude. (A) intertidal: log_e_(S)  =  −0.090+0.006 x latitude, r^2^ = 0.10, p = 2%; (B) subtidal: log_e_(S) = 0.067+0.007 x latitude, r^2^ = 0.08, p = 3%.
(EPS)

Table S1 Summary information on decapod collection sites among identified ecoregions. Tidal region indicates the sector that was sampled (intertidal and/or subtidal); quadrat sizes used are 100x = 1 m^2^ and 16x = 0.0625 m^2^, those listed without brackets with decapod records.
(DOCX)
