# Supplementary material for: Aspects of Benthic Decapod Diversity and Distribution from Rocky Nearshore Habitat at Geographically Widely Dispersed Sites
Source: PLoS One. 2011 Apr 14;6(4):e18606. doi: 10.1371/journal.pone.0018606 (PMC3077369; doi:10.1371/journal.pone.0018606)
Supplement: Table S1 — Summary information on decapod collection sites among identified ecoregions. Tidal region indicates the sector that was sampled (intertidal and/or subtidal); quadrat sizes used are 100x = 1 m2 and 16x = 0.0625 m2, those listed without brackets with decapod records. (DOCX) [file pone.0018606.s004.docx]

**Supplementary Table S1**

| **Site** | **Year** | **State/Province, Area** | **Country** | **Ocean** | **Tidal region** | **Quadrat size** | **Lat.** | **Long.** | **Ecoregion** |
| --- | --- | --- | --- | --- | --- | --- | --- | --- | --- |
| E2 | 2005 | Alaska, Boulder Patch | USA | Arctic | subtidal | 16x | 70.3180 | -147.7150 | Arctic |
| DSE | 2005 | Alaska, Camden Bay | USA | Arctic | subtidal | 16x | 70.0258 | -145.2588 | Arctic |
| Ridley Island | 2007 | British Columbia, Prince Rupert | Canada | Pacific | intertidal, subtidal | 100x | 54.2205 | -130.3297 | Brit. Columbia |
| Bath Island | 2006 | British Columbia, Vancouver | Canada | Pacific | intertidal, subtidal | 100x | 49.1449 | -123.6731 | Brit. Columbia |
| Cohen Island | 2003 | Alaska, Kachemak Bay | USA | Pacific | intertidal, subtidal | (100x), 16x | 59.5472 | -151.5472 | Gulf of Alaska |
| Elephant Island | 2003 | Alaska, Kachemak Bay | USA | Pacific | intertidal, subtidal | (100x), 16x | 59.5472 | -151.5139 | Gulf of Alaska |
| Outside Beach | 2003 | Alaska, Kachemak Bay | USA | Pacific | intertidal, subtidal | (100x), 16x | 59.4736 | -151.8942 | Gulf of Alaska |
| Akhiok Bay | 2003 | Alaska, Kodiak Island | USA | Pacific | intertidal, subtidal | (100x), 16x | 56.9465 | -154.1293 | Gulf of Alaska |
| Old Harbor | 2003 | Alaska Kodiak Island | USA | Pacific | intertidal, subtidal | (100x), 16x | 57.1567 | -153.3887 | Gulf of Alaska |
| Uyak Bay | 2003 | Alaska, Kodiak Island | USA | Pacific | intertidal, subtidal | (100x), 16x | 57.5736 | -154.1119 | Gulf of Alaska |
| Green Island | 2003 | Alaska, Prince William Sound | USA | Pacific | intertidal, subtidal | (100x), 16x | 60.3000 | -147.4122 | Gulf of Alaska |
| Knight Island | 2003 | Alaska, Prince William Sound | USA | Pacific | intertidal, subtidal | (100x), 16x | 60.4844 | -147.7356 | Gulf of Alaska |
| Montague Island | 2003 | Alaska, Prince William Sound | USA | Pacific | intertidal, subtidal | (100x), 16x | 60.3908 | -147.1217 | Gulf of Alaska |
| Kesen-numa | 2006 | Miyagi, Tohoku | Japan | Pacific | intertidal | 16x | 38.8968 | 141.6250 | Japan |
| Sakamoto | 2008 | Miyagi, Tohoku | Japan | Pacific | intertidal | (100x), 16x | 38.6455 | 141.4773 | Japan |
| Maenohama | 2006 | Okinawa, Akajima | Japan | Pacific | subtidal | 16x | 26.1865 | 127.2806 | Japan |
| Suzaki | 2008 | Tokyo, Chichi Island | Japan | Pacific | intertidal, subtidal | 16x | 27.0737 | 142.1885 | Japan |
| Hon Chong | 2009 | Khanh Hoa, Nha Trang | Vietnam | Pacific | intertidal | 100x, 16x | 12.2715 | 109.2068 | Vietnam |
| Torre del Serpe | 2006 | Apulia/Otranto | Italy | Mediterranean | intertidal, subtidal | (100x), 16x | 40.1447 | 18.5060 | Mediterranean |
| Looe | 2007 | Cornwall | UK | Atlantic | intertidal | 100x, (16x) | 50.3408 | -4.4595 | United Kingdom |
| Batten Bay | 2007 | Devon | UK | Atlantic | intertidal | 100x, (16x) | 50.3566 | -4.1269 | United Kingdom |
| Simpsons Island | 2007 2008 | New Brunswick, Passamaquoddy Bay | Canada | Atlantic | intertidal, subtidal | 100x, 16x | 45.0037 | -66.9135 | Canadian Atlantic |
| Canso | 2008 | Nova Scotia/Northeastern Shore | Canada | Atlantic | intertidal, subtidal | (100x), 16x | 45.3231 | -60.9640 | Canadian Atlantic |
| Birch Island | 2007 2008 | Maine/Cobscook Bay | USA | Atlantic | intertidal, subtidal | 100x, 16x | 44.8711 | -67.1493 | Canadian Atlantic |
| Playa de 16 | 2006 | Havana, Miramar | Cuba | Atlantic | intertidal, subtidal | 100x, (16x) | 23.1283 | -82.4222 | Caribbean |
| Fort Granby | 2007 | Southeast | Tobago | Atlantic | intertidal | (100x), 16x | 11.1862 | -60.6606 | Caribbean |
| Piedra Ahogada | 2008 | Anzoategui, Mochima | Venezuela | Atlantic | intertidal, subtidal | 100x | 10.3891 | -64.3478 | Caribbean |
| Punta Cruz | 2008 | Anzoategui, Mochima | Venezuela | Atlantic | intertidal, subtidal | 100x | 10.3957 | -64.3675 | Caribbean |
| Punta Tigrillo | 2007 | Anzoategui, Mochima | Venezuela | Atlantic | intertidal, subtidal | (100x), 16x | 10.3804 | -64.3951 | Caribbean |
| Boca Seca | 2008 | Falcón, Morrocoy | Venezuela | Atlantic | intertidal, subtidal | 100x | 10.8319 | -68.2408 | Caribbean |
| Cayo Mero | 2008 | Falcón, Morrocoy | Venezuela | Atlantic | intertidal, subtidal | 100x, 16x | 10.8198 | -68.2477 | Caribbean |
| **Site** | **Year** | **Area** | **Country** | **Ocean** | **Tidal region** | **Quadrat size** | **Lat** | **Long** | **ecoregion** |
| Playa Caimán | 2008 | Falcón, Morrocoy | Venezuela | Atlantic | intertidal, subtidal | 100x | 10.8517 | -68.2363 | Caribbean |
| Oeste San José | 2008 | Falcón, San José de la Costa | Venezuela | Atlantic | intertidal | 100x | 11.4465 | -68.8851 | Caribbean |
| Playa Chica | 2008 | Buenos Aires, Mar del Plata | Argentina | Atlantic | Intertidal, subtidal | 16x | -38.0200 | -57.5200 | Argentina |
| Pearly Beach | 2007 | Bantams Klip | South Africa | Atlantic | intertidal | (100x), 16x | -34.5848 | 19.4572 | Africa |
| Kizimkazi | 2007 | Menai Bay | Tanzania | Indian | intertidal | 100x, 16x | -6.4364 | 39.4664 | Africa |

**A**

**B**

**Supplementary Figure S1.**

**C**

**Supplementary Figure S2.**

**A**

**B**


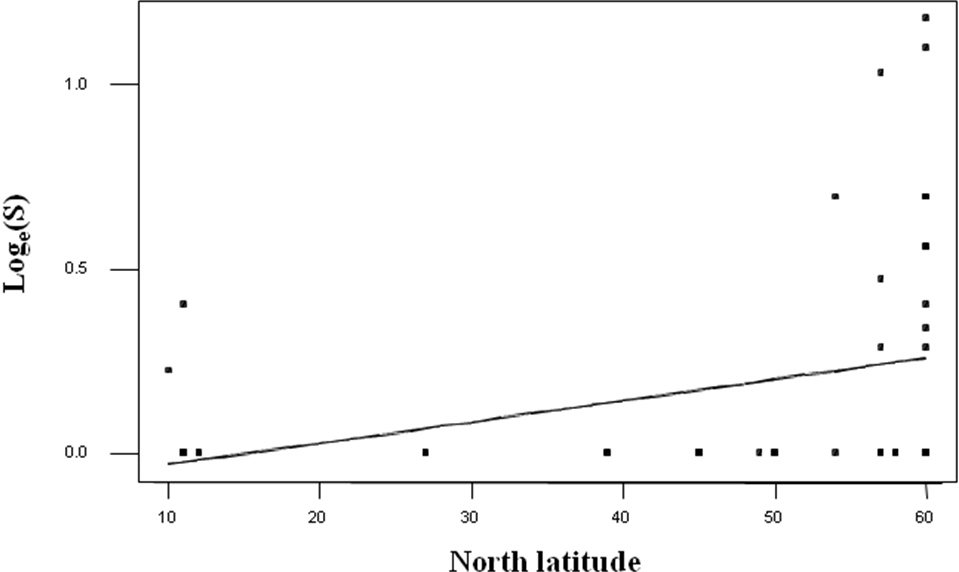


**A**


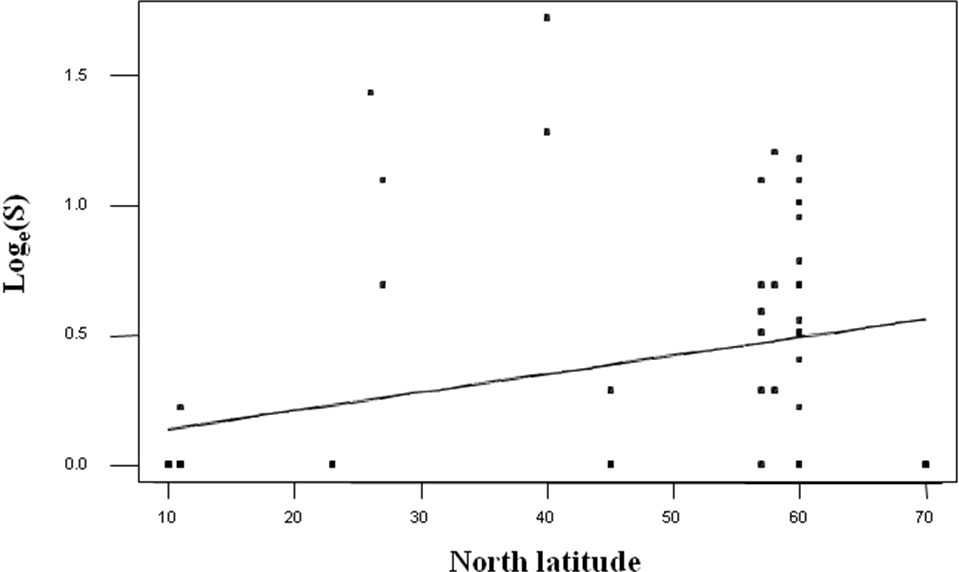


**B**

**Supplementary Figure S3.**
